# Protective Effects of Sheng-Mai-San on Right Ventricular Dysfunction during Chronic Intermittent Hypoxia in Mice

**DOI:** 10.1155/2016/4682786

**Published:** 2016-03-17

**Authors:** Cheng-Zhi Chai, Wei-Lan Mo, Xian-Fei Zhuang, Jun-Ping Kou, Yong-Qing Yan, Bo-Yang Yu

**Affiliations:** ^1^Department of Complex Prescription of TCM, China Pharmaceutical University, Nanjing, Jiangsu 211198, China; ^2^Jiangsu Provincial Key Laboratory for TCM Evaluation and Translational Research, China Pharmaceutical University, Nanjing, Jiangsu 211198, China

## Abstract

Right ventricular (RV) dysfunction and failure contribute to the increasing morbidity and mortality of cardiovascular diseases; however, current treatment strategies are grossly inadequate. Sheng-Mai-San (SMS) has been used to treat heart diseases for hundreds of years in China, and its protective effects on RV have not been observed. The present study was to investigate the protective effects of SMS aqueous extract on RV dysfunction in chronic intermittent hypoxia (CIH) mice model. The results showed that CIH mice model presented RV dysfunction and maladaptive compensation after 28-day-CIH and SMS treatment significantly reversed these changes. Diastolic function of RV was restored and systolic dysfunction was attenuated, including elevation of RV stroke volume and fractional shortening, as well as pulmonary circulation. Structurally, SMS treatment inhibited RV dilation, cardiomyocytes vacuolization, ultrastructure abnormalities, and the expression of cleaved caspase-3. Of importance, SMS showed remarkable antioxidant activity by decreasing the levels of malondialdehyde (MDA) and 4-hydroxynonenal (4-HNE), increasing the levels of superoxide dismutase (SOD) and heme oxygenase-1 (HO-1), as well as inhibiting the overexpression of 3-NT in RV. Our results indicate that SMS preserve RV structure and function in CIH-exposed mice by involving regulation in both ROS and Reactive Nitrogen Species (RNS) production.

## 1. Introduction

Impaired right ventricular (RV) function is associated with the increasingly increased morbidity and mortality of cardiovascular diseases and plays an important role in rising global health problems [1–4]. According to the results of clinical studies, patients with RV failure on the basis of other heart problems, such as left heart failure [5], acute myocardial infarction [6], or pulmonary hypertension and embolus [7–9], tend to suffer from inferior prognosis. So far, there are inadequate treatment strategies for RV dysfunction and decompensation [10].

Sheng-Mai-San (SMS) is a well-known Chinese herbal formula, which has over eight hundred years of application history in China. It is composed of Panax ginseng, Ophiopogon japonicus, and Schisandra chinensis and has been proved to be beneficial to Deficiency of both Qi and Yin Syndrome (DQYS), which is closely related to heart and lung diseases [11, 12]. An increasing number of studies have reported that it is beneficial to improve life quality and prolong life-span of the patients with pulmonary heart diseases through long-term administration of SMS [13–15]. However, the protective mechanisms of SMS on chronic dysfunctional RV have not been clarified.

It has been reported that people exposed to high altitudes for long term are susceptible to RV dysfunction [16, 17], and such pathological process can be simulated by experimental animals induced by chronic intermittent hypoxia (CIH) [18, 19]. Our group member has also demonstrated that CIH mice can simulate the main clinical features of DQYS [20] and has the characteristics of pulmonary heart diseases (unpublished). Several risk factors are involved in impairing RV function [21–23], and oxidative stress, the primary injury inflicted by CIH [24], is regarded as the crucial factor giving rise to RV decompensation and maladaptive compensation [25, 26]. Considering the beneficial effects of SMS on cardiac diseases, together with the reports of its antioxidant activity [11, 27], the present study was designed to test the hypothesis that SMS exerts protective effects on RV impairment through suppression of oxidative stress.

## 2. Materials and Methods

### 2.1. Preparation of SMS Aqueous Extract and Its Quality Control

The herbal materials of SMS were purchased from Nanjing Traditional Chinese Medicine Out-Patient Department in Nanjing, Jiangsu, and were identified by Professor Chun-gen Wang of Nanjing University of Chinese Medicine. A voucher specimen was deposited in Department of Complex Prescription of TCM, China Pharmaceutical University. The extraction procedure of SMS was carried out as previously described in our published literature [28]. Briefly, the three ingredients, including Radix ginseng (60 g), Radix ophiopogonis (180 g), and Fructus schisandrae (90 g), were mixed together and immersed in 10-fold, 8-fold, and 6-fold volumes of water (1 : 10, 1 : 8, and 1 : 6 w/v) to decoct for 1 h at 100°C, respectively. After filtrating with 8 layers of gauze, the three extractions were combined and concentrated to approximately 100 mL and stored at −20°C. SMS sample was then diluted by double distilled water to the required concentrations of oral administration before use. A high-performance liquid chromatography (HPLC) fingerprint analysis method has been established in our laboratory [28] and a HPLC-DAD-MS/MS analysis was used to identify the main constituents of SMS extracts referring to the published method of quality control in the present study.

### 2.2. Animal and Experimental Protocol

All animal welfare and experimental procedures complied with Chinese Institutional regulations. The experimental protocols were approved by the Animal Ethics Committee of the School, Chinese Materia Medica, China Pharmaceutical University. Eight-week-old male ICR mice (25-26 g) were purchased from Experimental Animal Center of Yangzhou University (Yangzhou, Jiangsu, China). All animals were housed in a temperature (23 ± 1°C), humidity (30%–40%), and light controlled (12 h light/dark cycle) room with food and water* ad libitum*.

Mice were randomly divided into four groups (10–12 mice per group) and received distilled water (control and model groups) or SMS (1.1 g/kg for SMS1 group and 5.5 g/kg for SMS2 group). In addition to the control group, all groups were exposed to chronic intermittent hypoxia (CIH) (nadir 7% to peak 8% oxygen, 20 min per day) in a chamber. All animals were sacrificed after echocardiography examination at the time of 28 days. Tissue samples were rapidly excised and stored at −70°C refrigerator.

### 2.3. Echocardiography

Mice were anesthetized by 4% chloral hydrate (0.1 mL/kg, i.p.) before echocardiography by Vevo2100 imaging system (VisualSonics Inc., Toronto, ON, Canada) with a 30 MHZ probe. Stable images were obtained in M, B, and Doppler Mode. RV inner dimension (RVID), RV stroke volume (RVSV), RV fractional shortening (RVFS), tricuspid valve early and late diastolic filling velocities (TV E/A ratio), pulmonary arterial velocity time integral (PA-VTI), pulmonary arterial preejection time (PA-PET), and pulmonary arterial ejection time (PA-ET) were measured.

### 2.4. Histology

Heart sections from formalin-fixed and paraffin-embedded tissues were prepared at 5 *μ*m thickness using a routine procedure. Tissue sections were stained with hematoxylin/eosin for general histology. A morphological analysis was used for semiquantitatively determining the extent of RV injury. Briefly, 5 visions (upper left, lower left, upper right, lower right, and middle) were observed under low magnification per section. Images were acquired by DFC 450C light microscope (Leica Microsystems Ltd., Wetzlar, Germany). The pathologist was unaware of the group assignment of individual mice.

### 2.5. Electron Microscopy

Right ventricle sample was fixed in paraformaldehyde (4%) solution (with 2.5% glutaraldehyde) as the previously described method [29, 30]. After 24 h, the tissue was sliced to prepare ultrathin samples to assess ultrastructure. Images were acquired by JEM-1001 transmission electron microscope (JEOL Ltd., Tokyo, Japan).

### 2.6. Enzyme-Linked Immunosorbent Assay (ELISA)

RV samples for ELISA analysis were prepared following the manufacturer' instructions. Expressions of malondialdehyde (MDA), superoxide dismutase (SOD), heme oxygenase-1 (HO-1), and 4-hydroxynonenal (4-HNE) were detected by ELISA (Nanjing Jian Cheng Biotech Co. Ltd., Nanjing, China).

### 2.7. Immunohistochemistry

8 *μ*m sections were prepared from frozen hearts and mounted on coated slides. The sections were first incubated in blocking buffer (1% BSA in PBS containing 0.3% Triton-X-100) for 1 h and incubated with the primary antibody against cleaved caspase-3 (1 : 300, rabbit anticleaved caspase-3; Abcam, Cambridge, UK) and 3-nitrotyrosine (3-NT, 1 : 300, mouse anti-NT; Abcam, Cambridge, UK) for 24 h. The sections were then washed by PBS and incubated in DAB substrate. The tissue was finally counterstained with hematoxylin and xylene before being washed with ethanol. Images were collected by DFC 450C light microscope (Leica Microsystems Ltd., Wetzlar, Germany).

### 2.8. Statistical Analysis

All data were presented as mean ± standard error of mean (SEM). Differences among groups were measured by one-way analysis of variance (ANOVA) followed by Dunnett's test (Prism 5, GraphPad, CA, USA). A value of *P* < 0.05 was considered as statistically significant.

## 3. Results

### 3.1. Effects of SMS on General Parameters

As the results show, the death rate of model group is higher than that of SMS treatment groups (1.1 or 5.5 g/kg), and administration with SMS significantly enhanced the survival rate. Compared with the mice in control group, right ventricle weight (RVW) in model group markedly increased while pretreatment with SMS (1.1 or 5.5 g/kg) reversed this abnormal performance significantly (Table 1).

### 3.2. Effects of SMS on RV Function

**Figure 1:**
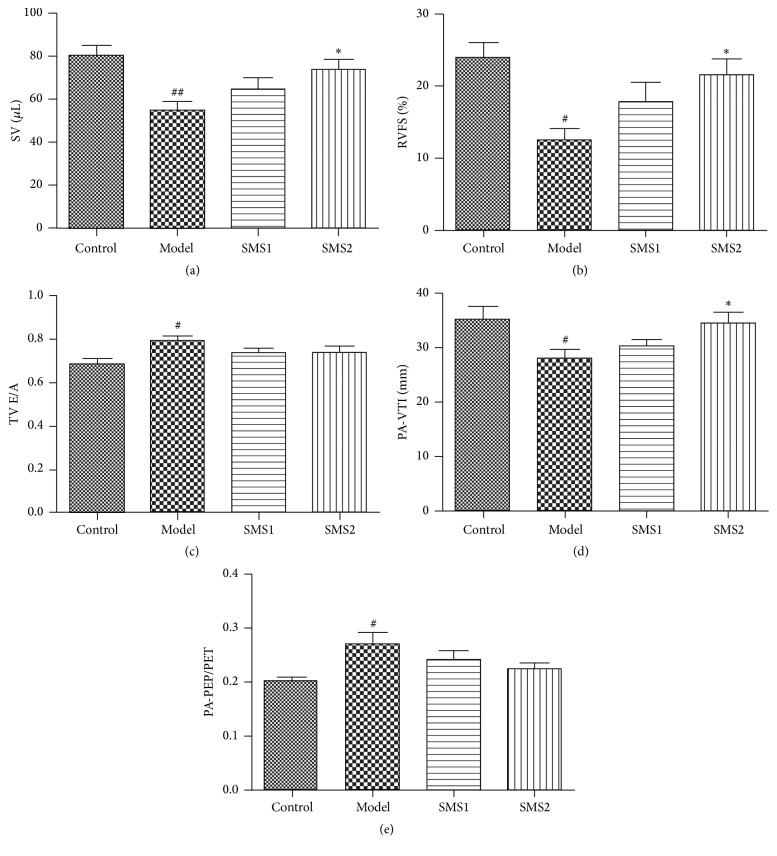
Echocardiographic assessment of RV function (*n* = 6). Functional parameters were SV (a), RVFS (b), TV E/A ratio (c), PA-VTI (d), and PA-PEP/PET (e). SMS1 (1.1 g/kg) and SMS2 (5.5 g/kg) prevented the occurrence of RV and pulmonary vascular dysfunction. SV, stroke volume; FS, fractional shortening; TV E/A, tricuspid valve early and late diastolic filling velocities; PA-VTI, pulmonary arterial velocity time integral; PA-PEP/ET, pulmonary arterial preejection time and ejection time. Values are presented as mean ± SEM; ^#^ *P* < 0.05 versus control, ^##^ *P* < 0.01 versus control, and ^*∗*^ *P* < 0.05 versus model.

RV function was evaluated by echocardiography. As the results show, right ventricular stroke volume (RVSV) and right ventricular fractional shortening (RVFS) of 28-day-CIH mice severely declined. In contrast, 4-week pretreatment with SMS (SMS2, 5.5 g/kg) prevented right ventricular from systolic dysfunction and the mice maintained normal levels of RVSV and RVFS (Figure 1, *P* < 0.05). In addition, CIH caused a significant rise in the tricuspid valve early and late diastolic filling velocities (TV E/A) ratio, which was prevented by administration of SMS, but without significance compared with the mice in CIH group (Figure 1, *P* < 0.05). Furthermore, the levels of pulmonary arterial velocity time integral (PA-VTI) of CIH mice significantly were reduced while the levels of pulmonary arterial preejection time and ejection time (PA-PEP/PET) were increased, which indicated the decline of pulmonary circulation function. Compared with the model group, SMS pretreatment preserved the normal levels of these two parameters indicating its effects on modulating pulmonary circulation function (Figure 1).

### 3.3. Effects of SMS on RV Structure

**Figure 2:**
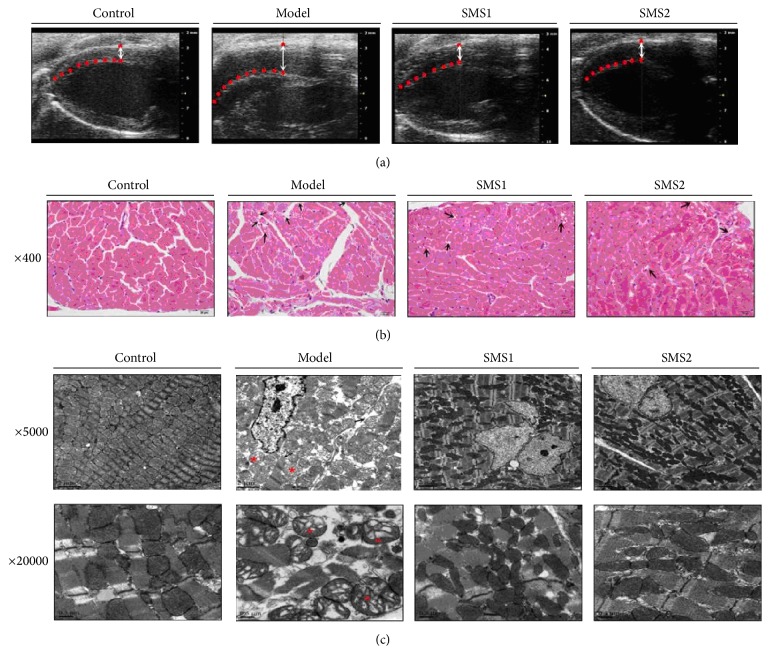
Beneficial effects of SMS on CIH-induced changes of RV structure in mice. RVID was determined by ultrasound ((a), *n* = 7). Dotted lines represented the interventricular septum. Histological examination of RV sections with H&E staining ((b), *n* = 3). Serve vacuolization was evidenced in 28-day-CIH mice (arrows shown). Compared with CIH mice, SMS2 (5.5 g/kg) treatment prevented this injury. Magnification, ×400; scale bar, 20 *μ*m. Myocardial ultrastructure examination in all groups by transmission electron microscopy ((c), *n* = 3). Images were acquired under magnification ×5000; scale bar, 2 *μ*m, and magnification, ×20000; scale bar, 0.5 *μ*m. SMS successfully improved disruption or dissolution of myofilaments, disordered sarcomere and swollen mitochondria (stars shown). Values are presented as mean ± SEM.

CIH induced a significant increase of RV inner dimension (RVID) compared with the control group (*P* < 0.05), and treatment with SMS (SMS2, 5.5 g/kg) prevented the increase of RVID markedly (Figure 2(a)). In addition, the alterations of RV were observed by light microscopic examination of RV sections stained with hematoxylin and eosin, immunohistochemistry, and transmission electron microscopy. Compared with the RVs of control mice, the RVs of CIH-treated mice showed a number of vacuoles in the cardiomyocytes (arrows shown) and a mild degree of inflammation (Figure 2(b)). Neither fibrosis nor necrosis occurred in all groups. The histopathological damage induced by CIH was minimal in mice that received SMS treatment. The ultrastructures of RV were shown at two magnifications (×5000; ×20000) and dissolution of myofilaments, disruption sarcomeres, and disarranged swollen mitochondria appeared with abnormal cristae (stars shown). SMS treatment protected against CIH-induced mitochondria damage.

**Figure 3:**
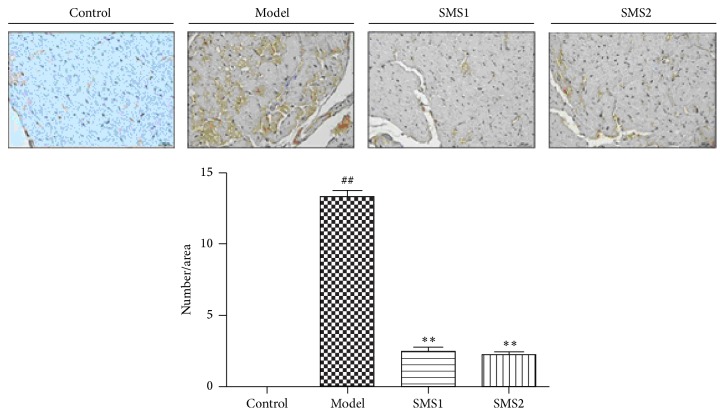
SMS reduced cleaved (active) caspase-3 expression in CIH-induced mice. Positive areas were brownish (arrows shown); magnification, ×400. Values are presented as mean ± SEM (*n* = 4); ^##^ *P* < 0.01 versus control and ^*∗∗*^ *P* < 0.01 versus model.

Then, we determined the levels of cleaved caspase-3 expression by immunohistochemistry (Figure 3). The results showed increased expression levels of cleaved (active) caspase-3 in CIH-treated mice and indicated that there existed apoptosis-like pathology in RV cardiomyocytes. SMS treatment presented lower levels of cleaved caspase-3 expression compared with the CIH group (SMS1 and SMS2 groups in Figure 3).

### 3.4. Effects of SMS on Oxidative Stress

**Figure 4:**
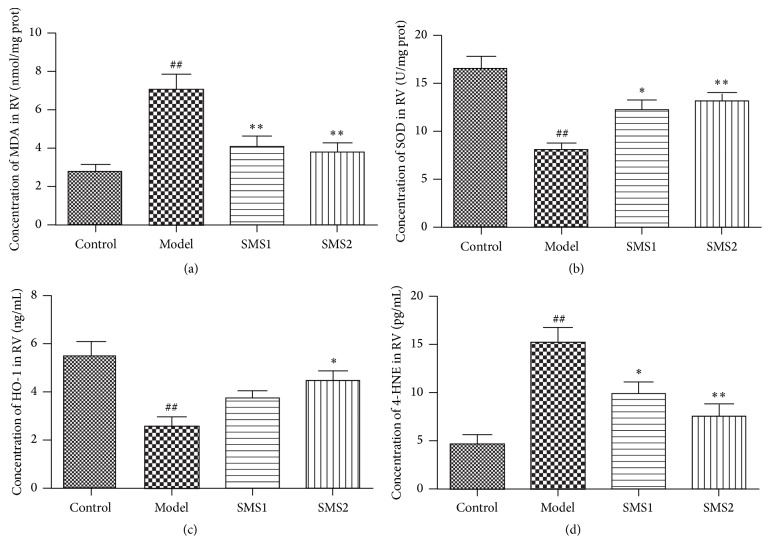
Influence of SMS on CIH-induced changes on malondialdehyde (MDA, (a)), superoxide dismutase (SOD, (b)), heme oxygenase-1 (HO-1, (c)), and 4-hydroxynonenal (4-HNE, (d)) in RV. Values are presented as mean ± SEM (*n* = 7-8) ^##^ *P* < 0.01 versus control; ^*∗*^ *P* < 0.05 versus model; ^*∗∗*^ *P* < 0.01 versus model.

**Figure 5:**
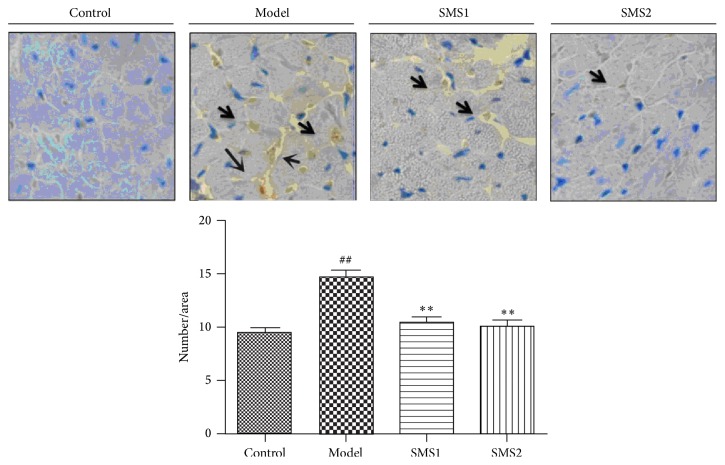
SMS reduced cardiac 3-nitrotyrosine (3-NT) expression in CIH-induced mice. Representative images are in 60 × 60 *μ*m squares (magnification, ×400). Positive stains of 3-NT are shown by arrows and quantitative analysis was used. Values are presented as mean ± SEM (*n* = 4), and five areas of each sample were observed, respectively. ^##^ *P* < 0.01 versus control; ^*∗∗*^ *P* < 0.01 versus model.

Cardiac oxidative stress was determined by enzyme-linked immunosorbent assay, as well as immunohistochemistry. As the results showed, the levels of MDA and 4-HNE in RV homogenates increased significantly, while SMS treatment completely prevented these alterations. Compared with the mice of CIH group, there were less decreases of SOD and HO-1 levels in SMS treatment groups (SMS1, SMS2), indicating the antioxidant properties of SMS (Figure 4). Additionally, we evaluated the levels of 3-NT in different groups through quantifying the positive stains in 60 × 60 *μ*m squares in five random areas of each group (Figure 5). The results showed that the positive points substantially increased in CIH-treated mice, while the positive points of SMS treatment (SMS1, SMS2) groups decreased significantly (Figure 5). The levels of 3-NT in four groups were consistent with the results of the oxidative stress factors MDA and 4-HNE.

## 4. Discussion

Deficiency of both Qi and Yin Syndrome (DQYS) is one of the common syndromes in cardiovascular diseases, and Sheng-mai San (SMS) is the representative prescription for the treatment of this syndrome. Experimental studies have proved that SMS can prevent heart ischemia, apoptosis, and so forth [31], which are in accord with its clinical application. In the present study, a chronic intermittent hypoxia (CIH) mice model [20] simulating the clinical features of DQYS was used, and the function and morphology of right ventricle (RV) in CIH-treated mice were furtherly observed, and the protective effects of SMS on impaired RV were also evaluated.

RV dysfunction and maladaptive remodeling are risk factors contributing to RV or left ventricular (LV) failure [1, 2] and are related to the increasing morbidity and mortality of cardiovascular diseases. In the present study, we found that RV remodeled in mice after 28-day-CIH (nadir 7% to peak 8% oxygen, 20 min per day) and the mortality of CIH-treated mice significantly increased, which were consistent with the reported references [32, 33]. By contrast, SMS administration attenuated systolic dysfunction by the elevation of RV stroke volume, fractional shortening, and pulmonary circulation. In addition, diastolic function of RV was also restored moderately as detected by echocardiography. The findings suggested that SMS exerted a protective effect on impaired RV induced by CIH.

Oxidative stress is regarded as an important pathophysiologic process of RV dysfunction and maladaptive compensation, although the exact mechanism of CIH giving rise to RV dysfunction is largely unknown [21, 25, 26]. In general, oxidative stress occurs from an imbalance between the formation of ROS and the antioxidant defense systems, and RNS also has a harmful effect on the cardiovascular system during this process. Substantial and persistent ROS and RNS will initiate signal pathways including apoptosis, inflammation, or fibrosis [23, 24]. There are correlative complex signal pathways between ROS and RNS of different origins in the heart. In the present study, we examined the activity of antioxidant enzymes except the content of lipid peroxidation products MDA and 4-HNE. As the results show, SMS remarkably reduced oxidative stress in RV by downregulation of the content of MDA and 4-HNE and upregulation of the activity of SOD and HO-1. Notably, overexpression of 3-NT in RV was also prevented by SMS administration. ROS and RNS in cardiomyocytes are derived from mitochondria [34, 35], NADPH oxidase [21, 36], xanthine oxidase [24], and uncoupled nitric oxide synthase [37, 38]. The results suggested that SMS is able to reduce oxidative stress by involving regulation of both ROS and RNS production.

The structure of the ventricular chamber is integral for optimal function and movement of blood flow in RV [22]. RV dilation reflected remodeling of the maladaptive decompensation processes within the context of lower cardiac cell regeneration [21, 39, 40]. CIH can make RV regress from compensation to decompensation [24]. In the short term, RV hypertrophy occurs to maintain the normal level of RV function [41]. However, RV hypertrophy will not be sustainable and becomes maladaptive decompensation over the long term. During long-term CIH exposure, remodeling and hypertrophy lead to adverse outcomes, including metabolic disorder, endothelial dysfunction, and excessive autonomous nervous system activity [24, 42, 43]. In the present study, histopathology examination of heart tissue demonstrated that CIH induced severe vacuolization, while necrosis or fibrosis has not been detected. Further transmission electron microscopy analysis showed swollen mitochondria, the cristae of mitochondria blurred, broken, or dissolved, and dissolution of myocardial myofilaments, suggesting that apoptosis might occur in RV. Next, we evaluated caspase-3 protein expression using western blotting analysis, which plays a central role in apoptosis [44]. The results showed that cleaved caspase-3 protein expression level of CIH-treated group significantly increased compared with that of the control group, further suggesting the feature of cardiomyocytes apoptosis. Compared with the model group, SMS prevented the occurrence of vacuolar cardiomyocytes and swollen mitochondria as well as downregulating caspase-3 protein expression, and the protective effects of SMS on cardiomyocytes apoptosis were confirmed.

PA-VTI can reflect RV ejection and systolic pressure when there is no obstruction in RV outflow tract [45, 46]. The ratio of PEP/ET is dependent on pulmonary arterial pressure, as well as the stiffness of RV [46]. Compared with the model group, SMS treatment (SMS2, 5.5 g/kg) restored pulmonary arterial function by regulating PEP/ET moderately and preventing the reduction of PA-VTI, which suggested that SMS mildly prevented the development of RV stiffness.

In conclusion, the present study showed that 28-day-CIH results in RV dysfunction and structural abnormalities while SMS administration can prevent these alterations from happening, possibly due to its antioxidant activity. The beneficial effects of SMS successfully preserved RV systolic ejection and prevented maladaptive compensation of RV, providing promising therapeutic strategies for the management of RV hypertrophy and failure in patients.

## Figures and Tables

**Table 1:** General parameters of mice (*n* = 8–10).

Group	Survivors/total mice	RVW, mg	RVW/HW, %
Control	10/10	45.0 ± 3.0	33.2 ± 2.4
Model	8/12	54.1 ± 2.1^#^	36.7 ± 2.4
SMS1	10/11	47.5 ± 2.3	33.1 ± 1.7
SMS2	10/10	46.4 ± 1.8	34.5 ± 1.7

RVW, right ventricular weight; RVW/HW, the ratio of right ventricular weight and heart weight; SMS1, SMS-treated group (1.1 g/kg); SMS2, SMS-treated group (5.5 g/kg). Values are presented as mean ± SEM. ^#^ *P* < 0.05 versus control.
